# MYH9 is an Essential Factor for Porcine Reproductive and Respiratory Syndrome Virus Infection

**DOI:** 10.1038/srep25120

**Published:** 2016-04-26

**Authors:** Jiming Gao, Shuqi Xiao, Yihong Xiao, Xiangpeng Wang, Chong Zhang, Qin Zhao, Yuchen Nan, Baicheng Huang, Hongliang Liu, Ningning Liu, Junhua Lv, Taofeng Du, Yani Sun, Yang Mu, Gang Wang, Shahid Faraz Syed, Gaiping Zhang, Julian A. Hiscox, Ian Goodfellow, En-Min Zhou

**Affiliations:** 1 Department of Preventive Veterinary Medicine, College of Veterinary Medicine, Northwest A&F University, Yangling, Shaanxi 712100, China; 2 Department of Basic Veterinary Medicine, College of Veterinary Medicine, Shandong Agricultural University, Taian, Shandong 271018, China; 3 State Key Laboratory of Veterinary Biotechnology, Harbin Veterinary Research Institute of Chinese Academy of Agriculture Science, Harbin 150001, China; 4 Department of Preventive Veterinary Medicine, College of Animal Science and Veterinary Medicine, Henan Agricultural University, Zhengzhou, Henan 450002, China; 5 Department of Infection Biology, Institute of Infection and Global Health, University of Liverpool, Liverpool L3 5RF, United Kingdom; 6 Department of Pathology, University of Cambridge, Addenbrooke’s Hospital, Hills Road, Cambridge CB2 2QQ, United Kingdom

## Abstract

Porcine reproductive and respiratory syndrome (PRRS) caused by the PRRS virus (PRRSV) is an important swine disease worldwide. PRRSV has a limited tropism for certain cells, which may at least in part be attributed to the expression of the necessary cellular molecules serving as the virus receptors or factors on host cells for virus binding or entry. However, these molecules conferring PRRSV infection have not been fully characterized. Here we show the identification of non-muscle myosin heavy chain 9 (MYH9) as an essential factor for PRRSV infection using the anti-idiotypic antibody specific to the PRRSV glycoprotein GP5. MYH9 physically interacts with the PRRSV GP5 protein via its C-terminal domain and confers susceptibility of cells to PRRSV infection. These findings indicate that MYH9 is an essential factor for PRRSV infection and provide new insights into PRRSV-host interactions and viral entry, potentially facilitating development of control strategies for this important swine disease.

Porcine reproductive and respiratory syndrome (PRRS) is a highly infectious porcine disease, and has become a global epidemic since its initial emergence in the US and Europe in the late 1980s, and early 1990s^1^,^2^,^3^, respectively. Due to causing severe reproductive failure and a high rate of late abortion and early farrowing in sows, and respiratory disease and mortality in young pigs^4^,^5^, PRRS is now widely recognized as one of the most economically significant diseases in the pork industry worldwide^6^.

The causative agent, PRRS virus (PRRSV), is a positive sense, single-stranded RNA virus, with a genome of approximately 15 kb in length. It is classified as a member of the *Arteriviridae* family, in the *Nidovirales* order^7^. Two major genotypes of PRRSV, European (type 1) and North American (type 2), have been identified based on their genetic and antigenic characteristics^8^. In addition, PRRSV appears to continue to evolve leading to the emergence of additional subgenotypes^9^,^10^, as well as the highly pathogenic PRRSV (HP-PRRSV) strains in China^11^,^12^,^13^.

The PRRSV genome encodes ten open reading frames (ORFs). The first two ORFs (ORF1a and ORF1b) encode replicase proteins whereas ORF2a, ORF2b, ORF3, and ORF4 encode four membrane-associated glycoproteins, designated as GP2a, E, GP3, and GP4, respectively. ORFs 5–7 encode three major structural proteins of the virion: the envelope glycoprotein GP5, the non-glycosylated membrane protein M, and the nucleocapsid protein N^14^,^15^. Recently, a novel structural protein GP5a was identified and encoded by newly identified ORF5a^16^.

PRRSV has a specific tropism for cells derived from the monocytic lineage, particularly differentiated macrophages such as porcine alveolar macrophages (PAMs)^17^. MARC-145 cells, derived from the African green monkey kidney cell line MA-104, are highly susceptible to PRRSV infection *in vitro*^18^,^19^. The limited tropism of PRRSV may at least in part be explained by the expression of certain cell-type specific receptors on host cells. Up to now, at least 6 molecules have been identified as PRRSV candidate receptors^20^. Among these molecules, CD163 has been shown to be indispensable for the infection^21^,^22^,^23^,^24^,^25^ and SIGLEC1 or CD169 has recently been confirmed as unrequired for PRRSV infection^26^. The scavenger receptor CD163 has been found to be a key factor for PRRSV infection. Expression of external CD163 protein in cells typically not susceptible to PRRSV confers the cell permissive^22^,^25^. Furthermore, the role of CD163 is proposed to be responsible for virus particles trafficking into early endosomes to facilitate a productive infection^23^, and PRRSV GP2a/4 glycoprotein complexes was identified to be interacting with CD163 for virus entry into susceptible host cell^24^. A recent study indicated that CD163 gene deleted pigs are fully protected from PRRSV infection^21^.

Previously, by using the anti-idiotypic monoclonal antibody (Mab2-5G2) to the idiotypic antibodies against the PRRSV GP5 protein, we identified a cellular protein with a molecular mass of 230 kDa in both MA-104 and PAMs interacting with Mab2-5G2^27^. In this study, we further demonstrated that this protein was identified as non-muscle myosin heavy chain 9 (MYH9) and that MYH9 was involved in PRRSV infection acting as an essential factor by the physical interaction with the GP5 protein of PRRSV via its C-terminal domain. MYH9 provided a bridge between the attachment of virus particles to cell surface receptors and the subsequent un-coating events required for genomic release within the host cell.

## Results

### Interaction of MYH9 with PRRSV GP5

Our previous study showed that the anti-idiotypic monoclonal antibody (Mab2-5G2) recognized a cellular protein in MA-104 cells and PAMs with the molecular mass of approximately 230 kDa^27^. Further analysis suggested that this protein was MYH9 (Supplementary Fig. 1a,b).

**Figure 1:**
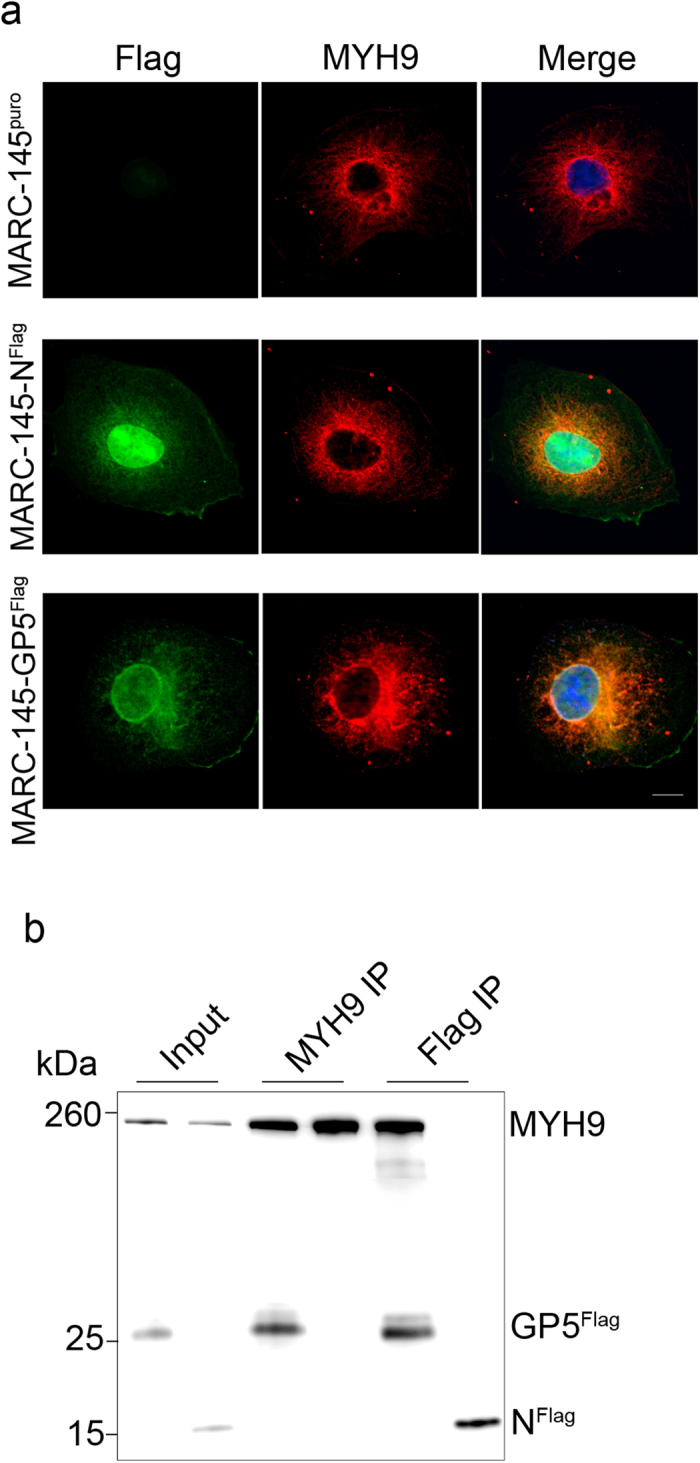
Interaction of GP5 with MYH9. (**a**) The cellular localization of the FLAG-tagged protein (green) or MYH9 (red) was imaged by confocal microscopy after staining with specific antibodies. DAPI counterstaining of nuclei (blue) is shown in merged images. Scale bars, 10 μm. (**b**) Co-immunoprecipitation of FLAG-tagged protein and MYH9 in MARC-145-N^Flag^ and MARC-145-GP5^Flag^ cell lysates using rabbit anti-MYH9 or rabbit anti-FLAG M2 antibodies. Co-IP products were analyzed by immunoblotting with mouse anti-FLAG M2 or Mab2-5G2 antibodies.

To confirm the interaction between GP5 and MYH9, MARC-145, PK-15, and COS-7 cell lines stably expressing FLAG-tagged GP5 (GP5^Flag^) were generated, as controls, MARC-145, PK-15, and COS-7 cell lines stably expressing the FLAG-tagged PRRSV nucleocapsid (N) protein (N^Flag^) were also constructed (Supplementary materials and methods; Supplementary Fig. 2). The location of GP5 and MYH9 in the cells was examined by confocal microscopy. In MARC-145 cells stably expressed GP5^Flag^, but not N^Flag^, was located together with MYH9 (Fig. 1a). The interaction between GP5 and MYH9 was confirmed by co-immunoprecipitation using anti-MYH9 or anti-FLAG antibodies. As shown in Fig. 1b, MYH9 co-precipitated with GP5^Flag^ in MARC-145-GP5^Flag^ cells, but not with N^Flag^ in MARC-145-N^Flag^ cells. In turn, GP5^Flag^ co-precipitated with MYH9 in MARC-145-GP5^Flag^ cell lysates, while N^Flag^ did not co-precipitate with MYH9 in MARC-145-N^Flag^ cell lysates. Taken together, these results indicate that GP5 was physically interacted with MYH9.

### MYH9 interacts with PRRSV at the early stage of virus infection

**Figure 2:**
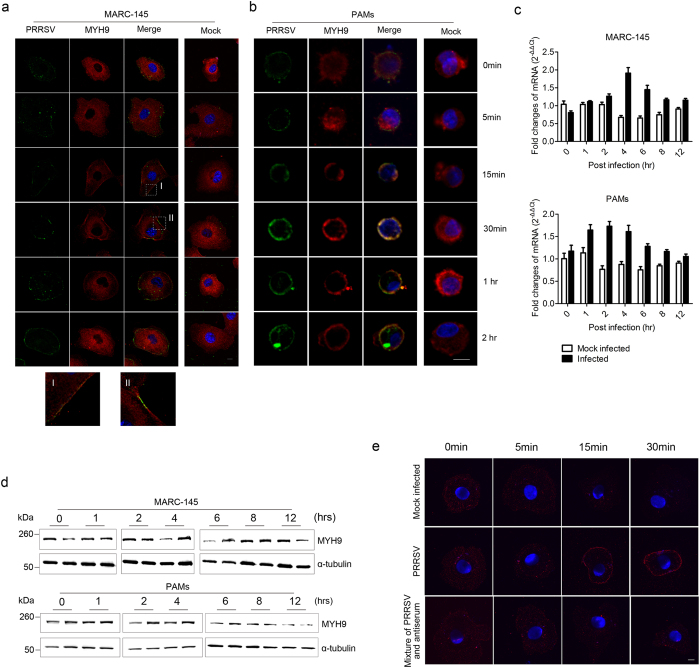
PRRSV co-located with MYH9 in the cells at early stage of PRRSV infection. MARC-145 cells (**a**) and PAM cells (**b**) were infected with PRRSV at an MOI of 100 at 4 °C for 1 hr, followed by a temperature shift to 37 °C for 0, 5, 15, 30 mins and 1, 2, 4, 6, 8, 12 hrs and then fixed, stained with rabbit anti-MYH9 and pig anti-PRRSV antibodies, and analyzed by confocal microscopy. The location of MYH9 (red) and PRRSV (green) in MARC-145 cells and PAMs at 4, 6, 8 and 12 hrs was shown in Supplementary Fig. 4a,b. Scale bars, 10 μm. (**c**) MYH9 mRNA relative level in PRRSV infected and mock infected MARC-145 cells and PAMs was tested by quantitative RT-PCR. The data was normalized to MYH9 mRNA level in mock infected cells at 37 °C for 0 hrs. Error bars, mean ± s.e.m. (n = 3). (**d**) MYH9 protein level in PRRSV infected and mock infected MARC-145 cells and PAMs was tested by Western blot using the indicated antibodies. The samples from left to right were PRRSV mock infected and infected cells lysates. The α-tubulin was tested as a loading control. (**e**) After PRRSV binding to MARC-145 cells at 4 °C for 1 hr, the cells were shifted to 37 °C for 0, 5, 15, and 30 min and fixed without permeabilization. MYH9 (red) localization was visualized by confocal microscopy after staining with specific antibodies. PRRSV pre-mixed with pig anti-PRRSV serum before infecting MARC-145 cells, and mock infected MARC-145 cells were used as the parallel controls. Nuclei (blue) were stained with DAPI. Scale bars, 10 μm.

PRRSV was incubated with MARC-145 cells and PAMs at MOI of 100 at 4 °C for 1 hr to allow the virus binding, followed by a shift to 37 °C to facilitate virus entry. At 0, 5, 15, 30 min and 1, 2, 4, 6, 8, 12 hrs after shifting to 37 °C, the cells were then fixed and the location of MYH9 and PRRSV were detected with the corresponding antibodies. PRRSV and MYH9 were located together in MARC-145 cells (Fig. 2a) and PAMs (Fig. 2b) between 15 min to 2 hrs after the cells were shifted to 37 °C, but not after 4 hrs post infection (Supplementary Fig. 3a,b). In addition, PRRSV infection lead to a significant increase in MYH9 mRNA and protein levels in MARC-145 cells and PAMs (Fig. 2c,d). These results confirmed that the interaction of PRRSV with MYH9 occurred at the early stage of virus infection and that the expression of MYH9 was stimulated by PRRSV infection.

MYH9 is normally localized in the cytoplasm of cells^28^, however, during the entry of Herpes Simplex Virus-1 (HSV-1) into cells, MYH9 is redistributed to the cell surface^29^. To investigate whether this redistribution also occurs during PRRSV infection, MARC-145 cells were inoculated with PRRSV (MOI 100) at 4 °C for 1hr to allow the virus binding, followed by a shift to 37 °C to facilitate virus entry. At 0, 5, 15, and 30 min after shifting to 37 °C, the cells were fixed with 4% paraformaldehyde and cell membrane-associated MYH9 were detected using anti-MYH9 antibody. As shown in Fig. 2e, at 0 and 5 min after temperature change, no MYH9 was detected on the plasma membrane of MARC-145 cells. However, a remarkable enrichment of MYH9 was observed on the plasma membrane at 15 and 30 min after the shift to 37 °C. However, when PRRSV premixed with pig anti-PRRSV serum to block the PRRSV attachment, MYH9 was not detected on the plasma membrane. These results suggest that PRRSV infection induced MYH9 redistribution from the cytoplasm to the cell surface similarly to that observed in HSV-1 infection and that MYH9 interacted with PRRSV during the early stage of virus infection.

### MYH9 is involved in PRRSV infection

**Figure 3:**
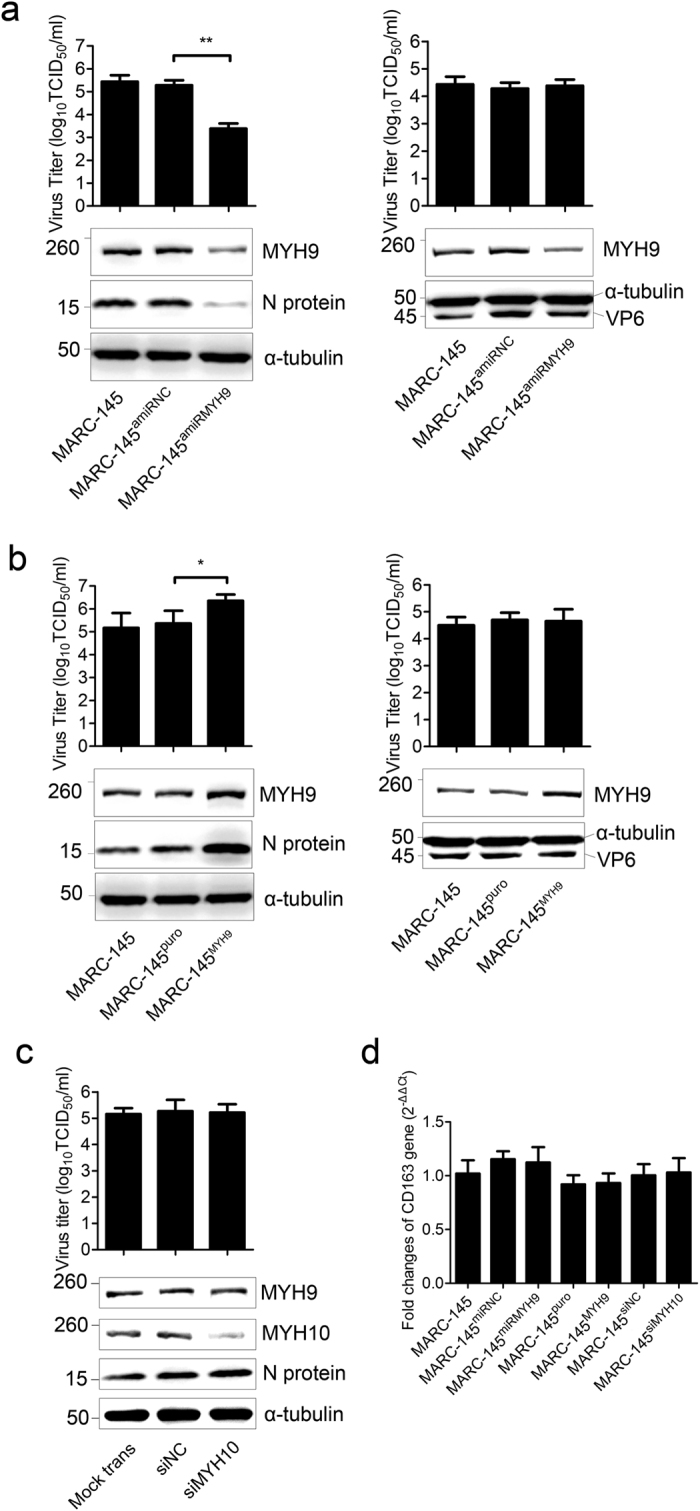
MYH9 expression level correlates with PRRSV infection. (**a**) TCID_50_ of PRRSV progeny virus titers in supernatant samples from cells with down-regulated MYH9 and western blot detection of MYH9 and PRRSV nuclear protein in the infected cells. The α-tubulin was tested as a loading control (left panel); TCID_50_ of pig rotavirus and western blot detection of MYH9 and pig rotavirus VP6 protein (right panel). (**b**) The effects of MYH9 overexpression on PRRSV (left panel) or pig rotavirus (right panel) replication in MARC-145 cells. (**c**) TCID_50_ of PRRSV progeny virus titers in supernatant samples from cells treated with MYH10 siRNA (siMYH9) or negative control siRNA (siNC) and Western blot detection of MYH9, MYH10 and PRRSV nuclear protein in the infected cells. (**d**) CD163 mRNA fold changes in MYH9 knockdown or overexpression cell lines, and in MYH10 knockdown cells. Error bars, mean ± s.e.m. (n = 3) *P < 0.05, **P < 0.01.

To evaluate whether MYH9 contributes to productive PRRSV infection, a MARC-145 cell line was engineered to stably express an artificial microRNA (amiRNA) against MYH9 (MARC-145^amiRMYH9^) (Supplementary Fig. 4a). The levels of MYH9 mRNA and protein in the MARC-145^amiRMYH9^ cells were significantly decreased compared to the control cells (Supplementary Fig. 4b,c) without any alteration of cell viability or growth kinetics compared to their parental cells (Supplementary Fig. 4d). The suppression of MYH9 expression significantly reduced PRRSV infection in MARC-145^amiRMYH9^ cells (Fig. 3a, left panel), but had no effect on rotavirus replication (Fig. 3a, right panel).

To further examine the role of MYH9 in PRRSV replication, a MARC-145 cell line was engineered to overexpress MYH9 (MARC-145^MYH9^) (Supplementary Fig. 4e,f). Overexpression of MYH9 markedly increased the yield of PRRSV (Fig. 3b, left panel), but did not affect the yield of porcine rotavirus (Fig. 3b, right panel).

Previous report demonstrated that MYH10 was detected in the MARC-145 cells^30^. To examine the association of PRRSV infection with MYH10 upon viral infection, small interference RNA (siRNA) targeting MYH10 (Supplementary Table 1) was applied to down-regulate MYH10 expression in MARC-145 cells. PRRSV infection assay result indicated that down-regulation of MYH10 did not affect virus production (Fig. 3c). To evaluate the effect of MYH9 or MYH10 expression on CD163 gene expression level in MARC-145, the mRNA fold changes of CD163 in MARC-145, MARC-145^amiRNC^, MARC-145^amiRMYH9^, MARC-145^puro^, MARC-145^MYH9^, MARC-145^siNC^ and MARC-145^siMYH10^ were tested by relative quantitative RT-PCR. The result showed that CD163 gene level did not change once the MYH9 or MYH10 expression was modulated (Fig. 3d). In addition, MYH9 C-terminal region protein (designated PRA^His^), but not MYH10 C-terminal region protein (designated PRB^His^), interacted with Mab2-5G2 (Supplementary material and method; Supplementary Fig. 1c). Taken together, these results demonstrated that MYH9, not MYH10, is involved in PRRSV infection.

### MYH9 is an essential factor in the association with CD163 facilitating susceptibility to PRRSV

**Figure 4:**
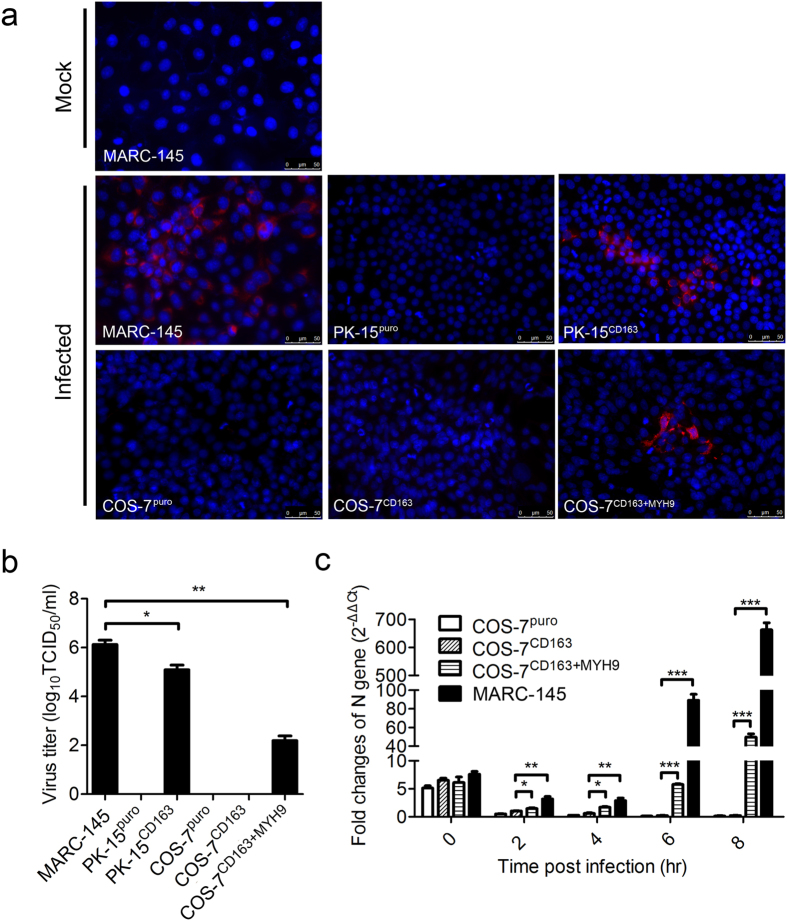
MYH9 is essential for PRRSV infection. (**a**) MARC-145, PK-15, PK-15^CD163^, COS-7, COS-7^CD163^, and COS-7^CD163+MYH9^ cell lines infected with PRRSV were fixed at 72 hrs post infection and imaged by confocal microscopy after staining with mouse antibodies against PRRSV N proteins (red). Nuclei (blue) were counter stained with DAPI. Scale bars, 50 μm. (**b**) TCID_50_ of progeny virus in the supernatant of the infected cells 72 hrs post infection. Error bars, mean ± s.e.m. (n = 3). *P < 0.05, **P < 0.01. (**c**) After allowing PRRSV bound to COS-7, COS-7^CD163^, COS-7^CD163+MYH9^, and MARC-145 cells at 4 °C for 1 hr, the cells were shifted to 37 °C for 0, 2, 4, 6 or 8 hrs. From the total mRNA, relative mRNA levels of PRRSV N gene were measured across the time course using quantitative RT-PCR. Relative folds of the N gene levels are shown in comparison with the N level in the COS-7^CD163^ cells at 37 °C for 2 hrs. Error bars, mean ± s.e.m. (n = 3).

Expression of CD163, a key receptor in PRRSV susceptibility, confers the unsusceptible cell lines to be PRRSV permissive^25^. PK-15 cells lack CD163 but express MYH9 (Supplementary Fig. 5a,b), while COS-7 cells lack both^31^ (Supplementary Fig. 5a,c). To further examine the potential role of MYH9 in PRRSV infection, PK-15 and COS-7 cells were engineered to express CD163 alone or both CD163 and MYH9 to generate PK-15^CD163^, COS-7^CD163^, and COS-7^CD163+MYH9^ cell lines (Supplementary Fig. 5d). Following PRRSV infection, PRRSV N protein could be detected in MARC-145, PK-15^CD163^, and COS-7^CD163+MYH9^ cells, but not in PK-15^puro^, COS-7^puro^, or COS-7^CD163^ control cells (Fig. 4a). At 72 hrs post infection, infectious PRRSV particles were also detected from the supernatant samples from MARC-145, PK-15^CD163^, or COS-7^CD163+MYH9^ cells, but not in PK-15^puro^, COS-7^puro^, or COS-7^CD163^ cell supernatant samples (Fig. 4b). The interaction of MYH9 with CD163 was examined using BiFC system (Supplementary materials and methods). The preliminary results showed that MYH9 C-terminal region (described below) interacted with CD163 tail domain (Supplementary Fig. 6).

Generally, newly produced infectious progeny virus is not detected until 8–12 hrs after PRRSV binding to host cells^32^. Therefore, the intracellular levels of the RNA encoding PRRSV N by qRT-PCR during the early phase of viral infection can be used to quantify viral binding to cells, virus uncoating and virus replication at the early stage of infection. The results showed that there were no differences in RNA levels of PRRSV N gene after PRRSV binding to cells at 4 °C. However, following shifting to 37 °C for 2 hrs, the level of PRRSV N RNA associated with the cells decreased than that at 0 hr, suggesting that not all bound virus can enter the target cells. The result also demonstrated that N gene level in COS-7 and COS-7^CD163^ cells was much lower than that in COS-7^CD163+MYH9^ and MARC-145 cells at 2 and 4 hrs after shifting to 37 °C, and at 6 and 8 hrs, the N gene level in COS-7^CD163+MYH9^ and MARC-145 cells was significantly increased (Fig. 4c). These findings indicated that MYH9 was not responsible for PRRSV binding to the cells, but the efficient PRRSV entry into COS-7 cells was depended on the expression of both CD163 and MYH9. Together, these results demonstrated that MYH9 present is essential for efficient PRRSV infection of the cells.

### The C-terminal region of MYH9 is involved in PRRSV infection

**Figure 5:**
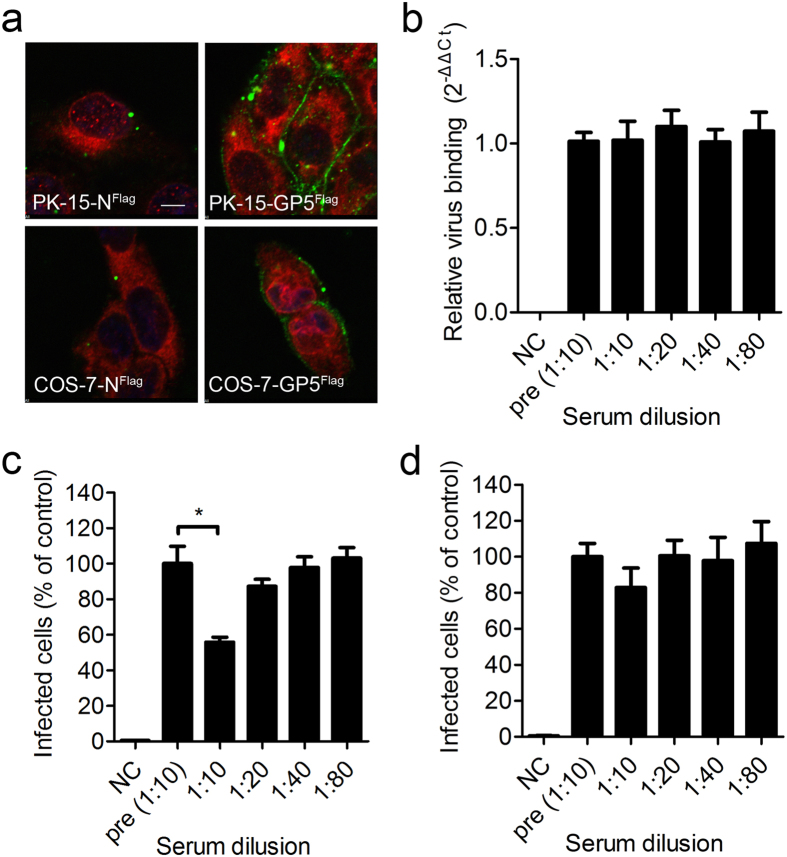
C-terminus of MYH9 is the binding domain of PRRSV GP5. (**a**) PK-15-N^Flag^, PK-15-GP5^Flag^, COS-7-N^Flag^, and COS-7-GP5^Flag^ cells were incubated with purified PRA^His^ at 4 °C for 1 hr, fixed after shifting to 37 °C for 30 min and imaged by confocal microscopy after stained with antibodies specific for His (green) and FLAG (red). Nuclei (blue) were counterstained with DAPI. Scale bars, 5 μm. (**b**) Anti-PRA^His^ serum dilutions were mixed with PRRSV-EGFP. The mixtures were incubated with MARC-145 cells at 4 °C for 1 hr, then cells were harvested and relative PRRSV binding levels were analyzed by quantitative RT-PCR. (**c**) After incubating with the mixtures at 4 °C for 1 hr, the cells were shifted to 37 °C for 30 min, and then washed to remove unbound virus. The quantity of PRRSV positive infected cells was analyzed by FACS 48 hrs post infection. Pre-immunization serum (pre) at 1:10 served as the control. (**d**) After cells were incubated for 1 hr at 4 °C in the mixtures, the cells were washed with DMEM prior to a shift to 37 °C for 48 hrs. The number of PRRSV positive infected cells was analyzed by FACS. Pre-immunization serum (pre) at 1:10 served as the control. In both (**c**) and (**d**) the data were shown as relative percentages compared to the value of the pre-immunization serum sample, whose mean value was set as 100% infection. Error bars, mean ± s.e.m. (n = 3). *P < 0.05.

To investigate whether GP5 interacts with the C-terminal domain of MYH9, purified PRA^His^ protein (supplementary Fig. 7a,b) was used to probe the cells expressing GP5^Flag^ or N^Flag^. As shown in Fig. 5a, PRA^His^ bound to PK-15-GP5^Flag^ or COS-7-GP5^Flag^, but not PK-15-N^Flag^ or COS-7-N^Flag^ control cells, indicating that a GP5 binding site was located in the C-terminal domain (aa1651–1960) of MYH9.

To evaluate the effects of PRA^His^ on PRRSV infection, rabbit anti-PRA^His^ serum was generated (Supplementary Fig. 7c). The recombinant PRRSV-EGFP strain^33^ was pre-mixed with anti-PRA^His^ serum and then incubated with MARC-145 cells for 1 hr at 4 °C to allow the virus binding. Quantitative RT-PCR demonstrated that incubation with anti-PRA^His^ serum did not block initial virus binding (Fig. 5b). However, when the cells were shifted to 37 °C and incubated for 30 min before washing out the virus/antisera mixture, the anti-PRA^His^ serum did inhibit PRRSV infection of MARC-145 cells in a dose-dependent manner (Fig. 5c). In contrast, when the mixture was washed out before the cell culture was shifted to 37 °C, the anti-PRA^His^ serum had no effect on viral infection (Fig. 5d). These results indicated that antiserum to MYH9 blocked PRRSV infection at a post binding step, most likely during the entry and/or the uncoating phase of the viral life cycle.

### Blebbistatin inhibits PRRSV infection *in vitro* and *in vivo*

**Figure 6:**
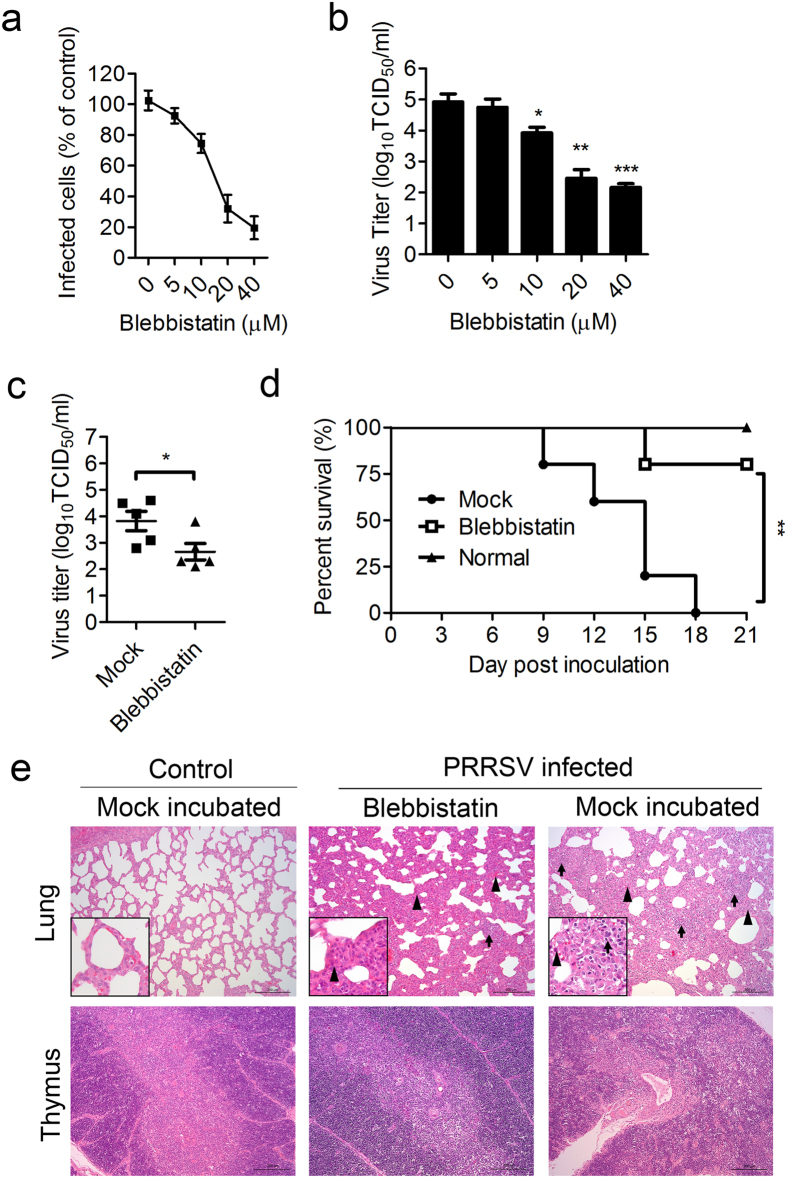
Blebbistatin inhibits PRRSV infection *in vitro* and *in vivo*. (**a**) MARC-145 cells were infected with PRRSV-EGFP in the presence of the indicated concentrations of blebbistatin. The cells were analyzed by FACS 48 hrs after infection. The data were shown as relative percentages compared to the value in the absence of blebbistatin, which the mean value was set at 100% infection. Error bars, mean ± s.e.m. (n = 3) (**b**) PAMs were infected with PRRSV in the presence of the indicated concentrations of blebbistatin. TCID_50_ of progeny virus titer was tested 24 hrs post infection. Error bars, mean ± s.e.m. (n = 3) (**c**) Virus titers in the serum samples of PRRSV infected pigs in PBS (mock) or blebbistatin treated groups 7 days after inoculation were determined by calculation of TCID_50_ on MARC-145 cells. Error bars, mean ± s.e.m. (n = 5 pigs per group) (**d**) Piglets received PBS (mock) or blebbistatin, and infected with PRRSV were monitored daily for mortality for 21 days. (**e**) Representative histopathological examinations for lung and thymus lesions stained by hematoxylin and eosin (H&E). Triangle indicates thickening of the alveolar septa or hyperaemia within alveolar septa. Arrow indicates inflammatory cells infiltrate. Scale bars, 200 μm. *P < 0.05, **P < 0.01, *** *P* < 0.001.

Blebbistatin, a specific inhibitor of myosin II ATPase activity^34^, was used to further invesigtate the role of MYH9 during PRRSV infection. Blebbistatin was found to inhibit PRRSV infection of MARC-145 cells and PAMs in a dose-dependent manner (Fig. 6a,b). The potential role of MYH9 on PRRSV infection was also investigated *in vivo* using a piglet model of infection. Piglets were either mock treated with PBS or treated with blebbistatin via intranasal delivery prior to PRRSV infection. Virus titers and piglet survival rates were then examined over a period of 21 days. Blebbistatin significantly reduced the viral load in serum at 7 days post infection (Fig. 6c). Moreover, blebbistatin treatment resulted in survival of 4/5 piglets, whereas 100% mortality was observed in piglets that did not receive blebbistatin (Fig. 6d). Piglets challenged with PRRSV demonstrated severe interstitial pneumonic lesions, decreased thymic lobule size, blurred boundaries between the thymic cortex and medulla, as well as decreased number of thymocytes in the medulla. In contrast, histological analyses indicated that the alveolar septa of lung were widened and mild pathological change was found in thymus with few thymocytes deleting in the medulla in PRRSV pigs that received blebbistatin (Fig. 6e). Taken together, these results indicated that MYH9 played an essential role in PRRSV infection both *in vitro* and *in vivo*.

## Discussion

Our previous work has shown that the anti-idiotypic monoclonal antibody, Mab2-5G2, generated against anti-PRRSV GP5 antibody recognized a 230 kDa protein from PRRSV permissive MA-104 cells and PAMs^27^. Anti-idiotypic antibody may bear a structural similarity to the GP5 itself^35^, therefore, the GP5 and Mab2-5G2 share common ability to interact with the cellular receptor of PRRSV^36^,^37^,^38^. Protein sequencing identified MYH9 as the potential binding partner of the anti-idiotypic antibody Mab2-5G2, suggesting that PRRSV GP5 may interact with MYH9. In this study, our results confirmed that PRRSV GP5 interacted with MYH9 in PRRSV permissive MARC-145 cells. Specifically, PRRSV GP5 was shown to interact with the C-terminal region of MYH9 (AA1651–1960) and this interaction occurred at the early stage of PRRSV infection.

MYH9 expression level apparently increased after PRRSV incubating with host cells, indicating that PRRSV infection was relevant with MYH9 expression level. Interference of MYH9 expression in PRRSV permissive cells affected virus progeny production but not pig rotavirus, suggesting the MYH9 expression level is an essential factor for productive PRRSV replication. HSV-1^29^ and severe fever with thrombocytopenia syndrome virus (SFTSV)^39^ have been shown to trigger rapid redistribution of MYH9 to the cell surface. The present study demonstrated the same pattern during PRRSV infection, possibly explaining why PRRSV infection can be inhibited by anti-PRA^His^ serum after the virus binding stage. This inhibition requires MYH9 redistribution to the cell surface, triggered by the entry of the virus, confirming that MYH9 interacts with GP5 after the virus binding stage.

Non-muscle myosin II 3 isoforms (MYH9, MYH10, and MYH14) exhibit a high level of amino acid sequence similarity except for their non-helical tail region^40^. Therefore, it has been proposed that different isoforms process redundant functions. As a HSV-1 entry receptor, MYH10 plays the same function as MYH9 for mediating HSV-1 entry by interacting with gB^41^. However, our results showed that MYH10 playing minimum role in PRRSV infection. It suggested that GP5 specifically interacts with MYH9.

Enveloped viruses enter cells through a series of steps, beginning with interactions between viral surface proteins and cell surface molecules. This is followed by the activation of cellular pathways that lead to membrane fusion between virus envelope and cell membrane, or internalization of the virus particles, as well as releasing of the virus genome to cytoplasm. Most viruses utilize multiple attachment factors, receptors, or entry mediators simultaneously to entry cell^42^,^43^. Previously, CD163 was identified as a major receptor in PRRSV infection. When CD163 was expressed in PRRSV non-permissive cells such as PK-15 and BHK-21 cell lines, these cells became susceptible to PRRSV infection^25^,^44^. Importantly, all of these cells express MYH9. On the other hand, COS-7 cells, expressing MYH10 but not MYH9, did not support PRRSV infection even with CD163 expression. This further confirmed that MYH10 was not relevant with PRRSV infection. Only when CD163 and MYH9 were co-expressed in COS-7 cells did they become permissive for PRRSV.

The binding of PRRSV was not different among the COS-7, COS-7^CD163^, and COS-7^CD163+MYH9^ cells, indicating that virus-cellular attachment is not dependent on CD163 or MYH9. The internalized virus assay result showed that some bound virus could enter COS-7^CD163+MYH9^ and MARC-145 cells after the cells were shifted to 37 °C for 2 hrs. It is known that CD163 is not responsible for PRRSV binding to cells^45^, but productive PRRSV infection requires trafficking through CD163-positive early endosomes^23^. PRRSV may need MYH9 to participate in the stage following entry. These results may also explain why PRRSV has a very restricted cell tropism as MYH9 is ubiquitously expressed in various mammalian tissues and cells types. In this study, the titer of progeny virus in COS-7^CD163+MYH9^ cells was much lower than that in MARC-145 or PK-15 cells, suggesting that virus replication levels may relate to the expression level of MYH9. On the basis of these results, we propose that PRRSV binding and entry into permissive cells takes place in three stages. Stage I: PRRSV binds to the cell surface with the help of other cellular proteins, such as heparin sulphate^46^ and sialoadhesin^47^. Stage II: After binding to the cell surface, the PRRSV GP2a/GP4 complex interacts with CD163^24^, and MYH9 redistributes to the cell surface to bind GP5 via a currently unknown mechanism. Stage III: MYH9 interacts with CD163^48^ and together support virus entry (Supplementary Fig. 6).

The blebbistatin inhibited PRRSV infection both *in vitro* and *in vivo* by preventing the contraction of MYH9 and may decrease the internalization of the virus into endosomes and may in turn prevent CD163 from moving to the cell surface to interact with the virus. This suggests that targeting the function of cellular factors critical for virus replication could be a successful therapeutic strategy. Currently, HSV-1^29^, Epstein-Barr virus^49^ and PRRSV (as reported in this study) have been known to utilize MYH9 as a functional receptor by interacting with virus specific protein. MYH9 has also been reported as a critical factor contributing to the efficiency of early infection of SFTSV *in vitro*^50^, and the activation of a Rho/NM II-dependent pathway facilitates Salmonella invasion^51^ or Sendai virus fusion with host cells^52^. The role of non-myosin II in infection of other pathogens remains an important area for further research.

## Materials and Methods

### Cells and Viruses

PK-15, MARC-145, 293T, and COS-7 cells were purchased from China Center for Type Culture Collection (CCTCC, Wuhan, China). MARC-145 and 293T cells were grown in Dulbecco’s minimal essential medium (DMEM) supplemented with 10% fetal bovine serum FBS (Gibco, Carlsbad, CA, USA). PK-15 and COS-7 cells were grown in minimal essential medium (MEM) supplemented with 10% FBS. PAMs were collected from PRRSV negative pigs (28 days old) and maintained in RPMI-1640 medium supplemented with 10% FBS. Wild-type highly pathogenic PRRSV (HP-PRRSV) SD16 strain was isolated by plaque purification and grown in MARC-145 cells as previously described^53^. HP-PRRSV SD16-EGFP recombinant virus was engineered as described previously^33^. Pig rotavirus (AV-55) was purchased from China Center of Veterinary Culture Collection and was propagated on MA-104 cells^54^.

### Reagents

Monoclonal mouse antibodies against PRRSV-N and CD163, and Mab2-5G2, polyclonal mouse antibodies against GP5, polyclonal rabbit antibodies against PRA^His^, and pig anti-PRRSV sera were produced and purified in house. Mouse antibodies against FLAG M2 (F3165), His (SAB4600386), and α-tubulin (T6074), rabbit antibodies against FLAG (F7425), MYH9 (M8064) and MYH10 (M7939) were purchased from Sigma-Aldrich (St. Louis, MO, USA). Mouse antibodies against Rotavirus [A2] (ab181695) was purchased from Abcam (Cambridge, MA, USA). Rabbit antibodies against GFP (AG279) were purchased from Beyotime (Jiangsu, China). Alexa Fluor 488 (115545003) or Cy3 (115165164) conjugated goat anti-mouse IgG antibodies, FITC (111095045) or Cy3 (111165003) conjugated goat anti-rabbit IgG antibodies, Cy3 (713165003) conjugated goat anti-swine IgG antibodies, and HRP conjugated goat anti-mouse IgG antibodies (115035003) were purchased from Jackson Immunoresearch (West Grove, PA, USA). 4,6-diamidino-2-phenylindole (DAPI) (P36931) was purchased from Life Technologies (Grand Island, NY, USA). X-tremeGENE HP DNA Transfection Reagent (06366236001), X-tremeGENE siRNA Transfection Reagent (04476093001), FastStart Universal SYBR Green Master (Rox) (04913850001), gel extraction, PCR purification, plasmid isolation, total RNA purification kit, and molecular biology reagents including restriction enzymes, and reverse transcription reagents were purchased from Roche (Basel, Switzerland). T4 DNA ligase, Taq DNA polymerase and In-Fusion HD Cloning Kit (639648) were purchased from Takara (Dalian, China). Isopropyl b-d-1-thiogalactopyranoside (IPTG) (R0392) was purchased from Thermo Scientific (St. Rockford, II, USA). Dynabeads Protein G (10003D) was purchased from Invitrogen (Grand Island, NY, USA). Blebbistatin (1760) was purchased from TOCRIS Bioscience (Houston, TX, USA).

### Immunoprecipitation Assays

First, Dynabeads Protein G was coupled to either rabbit anti-MYH9 or rabbit anti-FLAG M2 antibody according to the manufacturer’s protocol. The cells of interest were harvested, washed with PBS, and lysed in TNE buffer^29^ containing a proteinase inhibitor cocktail (Sigma). After centrifugation, the supernatant was collected and then incubated with the coupled beads for 2 hrs at 4 °C. Antigen bound beads were washed 3 times with TNE buffer. The bound proteins were then eluted with glycine buffer (pH = 3.0) and analyzed by immunoblotting.

### Virus Infection

To analyze the susceptibility of PRRSV to the different cell lines, the cells were inoculated with PRRSV at an MOI of 0.01 for 1 hr at 4 °C. After washing out unbound virus, the binding and replication levels of virus in different cell lines were quantified by real-time PCR for PRRSV N gene. At various time post infection, virus yields in the supernatant were measured by TCID_50_ assay in MARC-145 cells. For detecting the location of MYH9 or PRRSV at various time post infection, PRRSV at MOI of 100 (TCID50 = 10^6^/ml with 1.4 ml) was used to incubate with MARC-145 cells for 1 hr at 4 °C for synchronizing PRRSV binding to the cells. For detecting the inhibition of PRRSV infection in the cells, the percentage of GFP positive cells from PRRSV-EGFP infected cells were analyzed by FACS at 48 hrs post infection and normalized by control. Trypsin-activated rotavirus (30 min at 37 °C with 10 mg/ml trypsin) propagated from MA-104 cells was added to MARC-145 cells at an MOI of 0.01 and adsorbed at 4 °C for 1hr, then shifted to 37 °C to allow virus replication^54^.

### Inhibition of Virus Infection Assays

Rabbit anti-PRA^His^ sera and pre-immune sera were serially diluted and incubated with PRRSV-EGFP (MOI of 0.01). The mixture was added to MARC-145 cells in 24-well plates and incubated at 4 °C for 1 hr to allow virus binding to cells. After washing out the unbound virus, the virus bound to cells was analyzed by qRT-PCR. To analyze the effect of anti-PRA^His^ serum on PRRSV infection, two different experiments were set up. First, the virus-serum mixture was incubated with the cells at 4 °C for 1 hr to allow binding. The virus-serum mixture was not washed out until the cells were shifted to 37 °C and incubated for 30 min. The cells were then re-fed with the appropriate medium and 48 hrs after infection and analyzed by flow cytometry. Second, the virus-serum mixture was washed out before the cells were shifted to 37 °C.

For inhibition with blebbistatin, MARC-145 cells or PAMs were pre-treated with various concentrations of blebbistatin for 30 min and then inoculated with PRRSV-EGFP or PRRSV SD16 stain, respectively, at MOI of 0.01. After removal of the inoculum, the cells were re-fed with medium containing the same concentrations of blebbistatin. For MARC-145 cells, cells were analyzed by flow cytometry 48 hrs after the infection and for PAMs, TCID_50_ of progeny virus titer was measured 24 hrs after the infection.

### RNAi and Quantitative RT-PCR

Artificial microRNA expression vectors were transfected into the cells for down regulating MYH9 expression according to the protocol of X-tremeGENE HP DNA Transfection Reagent and the stably transfected cells were selected. siRNAs targeting MYH10 gene and negative control siRNA (Supplementary table 1 26–29) were transiently transfected into the cells according to the instruction of X-tremeGENE siRNA Transfection Reagent. Total RNA was extracted using High Pure RNA Isolation Kit (Roche). Relative mRNA levels of PRRSV N gene, MYH9 and CD163 were determined using primers 14–19 listed in Supplementary Table 1, and normalized to β-Actin mRNA levels, amplified using primers 20 and 21 listed in Supplementary Table 1. The quantification was performed in Step One Plus Real-Time PCR System (Applied Biosystems) using FastStart Universal SYBR Green Master.

### Indirect Immunofluorescence Assay

Cells were grown on top of microscope coverslips in 24-well tissue culture plates (801007; NEST). At each experimental endpoint, the cells were fixed with 4% paraformaldehyde for 10 min at room temperature (RT), and permeabilized with 0.2% Triton X-100 in PBS at RT for 10 min. The cells were subsequently blocked with 1% bovine serum albumin (BSA) in PBS for 30 min at RT and then incubated with the indicated primary antibodies for 2 hrs. After washing five times with PBS, the cells were incubated for 1 hr at RT with Fluor-labeled goat polyclonal antibodies: Alexa Fluor 488 or Cy3 conjugated goat anti-mouse IgG antibodies, FITC or Cy3 conjugated goat anti-rabbit IgG antibodies, Cy3 conjugated goat anti-swine IgG antibodies. Finally, the cells were counterstained with DAPI, and cell staining was visualized using A1R Resonant Scanning Confocal Microscope (Nikon) running NIS-Elements imaging software.

### Immunoblotting

Proteins were separated on a NuPAGE 4–20% gradient Bis-Tris gel (Invitrogen) and electro-transferred onto Immobilon-P membranes (Millipore). The membranes were blocked with 3% powdered skim milk (BD Biosciences) in TBS [10 mM Tris-HCl (pH 8.0), 150 mM NaCl] with 0.05% Tween-20 at 4 °C for 2 hrs, and then reacted with the primary antibody at RT for 2 hrs. The blots were subsequently incubated with horseradish peroxidase (HRP)-labeled goat anti-mouse IgG antibody (1:5000) for 2 hrs at RT. Finally, the proteins were visualized using enhanced chemiluminescence (ECL) reagents (Amersham Biosciences).

### Animal Experiments

Fifteen PRRSV-free piglets (28 days old) were obtained from a PRRS free farm in Yangling and randomly divided into three groups. Group 1 piglets (n = 5) received PRRSV only, Group 2 piglets (n = 5) were given blebbistatin and challenged with PRRSV, and Group 3 piglets (n = 5) were used as the negative control. The animals were kept in 3 separate rooms and fed a commercial diet and water ad libitum throughout the experiment. Group 1 and 3 piglets were given PBS (2 ml) and Group 2 piglets were given 200 μM of blebbistatin diluted in 2 ml of PBS intranasally (i.n.) per pig on experimental day (ED) 7 and 14. Group 1 and 2 piglets were challenged i.n. with PRRSV SD16 strain (7.5 × 10^5^ TCID_50_/pig) on ED 8, and Group 3 piglets received PBS. Serum samples were collected on 7^th^ day after the challenge from each piglet in Group 1 and 2, and virus titers were determined for TCID_50_. Piglets were monitored daily for 21 days, and the surviving piglets were euthanized 21 days post infection. Lung and thymus tissues were collected for the histopathological examinations. Animal experiments were performed according to Chinese Regulations of Laboratory Animals and the approval license number was NWAFU (Shan) 20131017/02, which was approved by the Animal Care and Use Committee of Northwest A&F University.

### Microscopic Pathological Examinations

Lung and thymus tissues were collected, fixed with 4% paraformaldehyde solution at room temperature for 48 h and then stained with hematoxylin and eosin (H&E) for observing pathological changes.

### Statistical Methods

Student’s T test was performed with a value of *P* < 0.05 considered statistical significant. ANOVA was used to identify significant group differences.

## Additional Information

**How to cite this article**: Gao, J. *et al*. MYH9 is an Essential Factor for Porcine Reproductive and Respiratory Syndrome Virus Infection. *Sci. Rep.* **6**, 25120; doi: 10.1038/srep25120 (2016).

## Supplementary Material

Supplementary Information
