# Supplementary material for: MYH9 is an Essential Factor for Porcine Reproductive and Respiratory Syndrome Virus Infection
Source: Sci Rep. 2016 Apr 26;6:25120. doi: 10.1038/srep25120 (PMC4845007; doi:10.1038/srep25120)
Supplement: Supplementary Information [file srep25120-s1.pdf]

MYH9 is an Essential Factor for Porcine Reproductive and Respiratory Syndrome Virus Infection

Jiming Gao, Shuqi Xiao, Yihong Xiao, Xiangpeng Wang, Chong Zhang, Qin Zhao, Yuchen Nan, Baicheng Huang, Hongliang Liu, Ningning Liu, Junhua Lv, Taofeng Du, Yani Sun, Yang Mu, Gang Wang, Shahid Faraz Syed, Gaiping Zhang, Julian A. Hiscox, Ian Goodfellow and En-Min Zhou

Supplementary information

Supplementary methods

Generation of Vectors and Expression Constructs

To generate the fusion protein containing His, the C-terminal domain of MYH9 (PRA^{His}) gene encoding amino acids aa1650-1960 and MYH10 (PRB^{His}) gene encoding amino acids aa1476-2006 were amplified by RT-PCR from mRNA in MARC-145 cells. The cDNA fragment was then cloned into pET-28a(+) (Novagen, Hornsby Westfield, Australia) using primers 1-4 listed in Supplementary Table 1. pTRIP-N^{Flag}-puro and pTRIP-$GP5^{Flag}$-puro were constructed using primers 5, 6, 7, 8 and 9 listed in Supplementary Table 1 for expression of PRRSV N and GP5 protein with a Flag tag. The pTRIP-CD163-puro plasmid was used to express swine CD163, which was cloned using fusion technology (Takara) from PAM total mRNA using primers 10 and 11 listed in Supplementary Table 1. The MYH9 open reading frame from MARC-145 cells total mRNA was cloned into the PiggyBac vector (System bioscience, Mountain View, CA, USA) by fusion technology (Takara) using primers 12 and 13 listed in Supplementary Table 1, and designated PB-MYH9-GFP+puro. Plasmid pcDNA6.2-$amiR^{MYH9}$-EmGFP, and pcDNA6.2-$amiR^{NC}$-EmGFP were used to generate stable cell lines expressing amiRNA against MYH9 or negative control amiRNA, which were constructed with the following oligonucleotides 5'-TGC TGA TAT ATT GCT GCA CTG AGT GTG TTT TGG CCA CTG ACT GAC ACA CTC AGC AGC AAT ATA T-3' and 5'-TGC TGA AAT GTA CTG CGC GTG GAG ACG TTT TGG CCA CTG ACT GAC GTC TCC ACG CAG TAC ATT T-3'. Plasmids PRA-VC, $CD163^{a}$-VN, $CD163^{b}$-VN, $CD163^{c}$-VN

and $CD163^{d}$-VN for BiFC assay were constructed by using the primers 30-37 listed in Supplementary Table 1.

Selection of Stable Cell Lines

PK-15^{CD163} and PK-15^{puro} cell lines were constructed as previously described[1] by transfecting PK-15 cells with PB-CD163-GFP-puro and PB-GFP-puro plasmids. Pseudo-typed lentivirus particles were generated according to the previously described method[2]. Briefly, a monolayer of HEK293T cells were transfected with expression plasmids (2 µg) (pTRIP-puro, pTRIP-N^{Flag}-puro, pTRIP-$GP5^{Flag}$-puro, or pTRIP-CD163-puro) with packing plasmids pXPAX2 (1 µg), and pMD2.G (1 µg), using X-tremeGENE HP DNA Transfection Reagent (Roche). Two days after transfection, supernatants were collected. MARC-145, PK-15, and COS-7 cells were transduced with the recombinant lentivirus collected from supernatants of transfected HEK293T cells. Stable cell clones were selected under pressure of puromycin (0.5 mg/ml) in maintenance medium. The puromycin-resistant cells transduced with recombinant lentivirus derived from pTRIP-N^{Flag}-puro, pTRIP-$GP5^{Flag}$-puro, pTRIP-CD163-puro, or pTRIP-puro were designated MARC-145-N^{Flag}, PK-15-N^{Flag}, COS-7-N^{Flag}, MARC-145-$GP5^{Flag}$, PK-15-$GP5^{Flag}$, COS-7-$GP5^{Flag}$, COS-7^{CD163}, MARC-145^{puro}, PK-15^{puro}, or COS-7^{puro} cells, respectively. The COS-$7^{CD163+MYH9}$ cell line was obtained by transfecting COS-7^{CD163} cells with plasmid PB-MYH9-GFP+puro, which were then subcloned following GFP selection. MARC-145 cells were transfected with pcDNA6.2-$amiR^{MYH9}$ or pcDNA6.2-$amiR^{NC}$ and selected with blasticidin (4.0 mg/ml) in maintenance medium. Resistant cells transduced with pcDNA6.2-$amiR^{NC}$ were designated MARC-145^{amiRNC} cells and used as a control. Single colonies transduced with

pcDNA6.2-amiR^{MYH9} were isolated and screened by immunoblotting with Mab2-5G2 and quantitative RT-PCR to generate the MARC-145^{amiRMYH9} cell line.

Bimolecular fluorescence complementation (BiFC) assay

To construct expression plasmids for BiFC assay, plasmid expressing Venus fluorescent protein (VFP) was generated based on plasmid pEYFP-N1 (BD Biosciences Clontech) as described previously[3]. 293T cells grown in 6 well plates were transiently co-transfected with combinations of plasmids CD163^{a}-VN (1 μg) and PRA-VC (1 μg), CD163^{b}-VN (1 μg) and PRA-VC (1 μg), CD163^{c}-VN (1 μg) and PRA-VC (1 μg), or CD163^{d}-VN (1 μg) and PRA-VC (1 μg) and incubated at 37 ℃ for 20 hrs. Fluorescence emission was detected using a fluorescence microscope and western blot analysis was performed to detect protein expression.

Generation of Rabbit anti-PRA Antisera

Two New Zealand white rabbits (from Medical Laboratory Animal Center, Xi'an Jiaotong University) were immunized with purified PRA^{His} (50μg/rabbit) mixed with QuickAntibody immunoadjuvant (KangBiQan, China) subcutaneously for 3 times in a 2-week interval. Fourteen days after the 3rd immunization, sera were collected and titrated by indirect ELISA. Animal experiments were performed according to Chinese Regulations of Laboratory Animals and the approval license number was NWAFU (Shan) 20140825/05, which was approved by the Animal Care and Use Committee of Northwest A&F University.

Reference

1 Wang, X. *et al.* PK-15 cells transfected with porcine CD163 by PiggyBac transposon system are susceptible to porcine reproductive and respiratory syndrome virus. *J Virol Methods* **193**, 383-390 (2013).

2 Du, E. & Tikoo, S. K. Efficient replication and generation of recombinant bovine adenovirus-3 in nonbovine cotton rat lung cells expressing I-SceI endonuclease. *J Gene Med* **12**, 840-847 (2010).

3 Nagai, T. *et al.* A variant of yellow fluorescent protein with fast and efficient maturation for cell-biological applications. *Nat Biotechnol* **20**, 87-90 (2002).

4 Gao, J., Kong, N., Xiao, H., Zhang, A. & Zhou, E. M. Development of Real-Time PCR Methods for the Detecttion of CD163 and Porcine Reproductive and Respiratory Syndrome Virus N Genes in Marc-145 cells. *J Ani Vet Adv* **11**, 4489-4493 (2012).

Supplementary figures

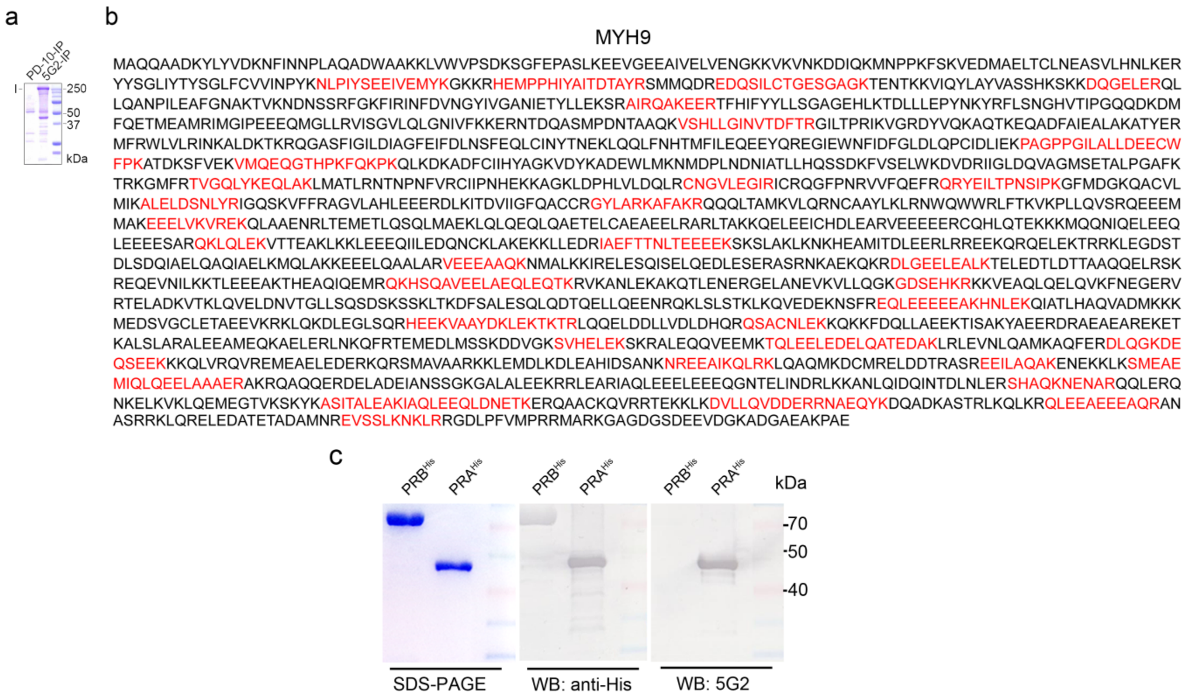

Supplementary Figure 1. Identification of MYH9. (a) Dynabeads protein G labeled with Mab2-5G2 or control IgG (PD-10) were incubated with MARC-145 cell lysate to perform immunoprecipitation. Immunoprecipitates were separated in a denatured gel and stained with Coomassie brilliant blue. Band I was analyzed by mass spectrometry and identified as MYH9. **(b)** The amino acid sequence of MYH9 in MARC-145 cells was determined by mass spectrometry analysis. The 34 peptide sequences identical to MYH9 are shown in red. (**c**) MYH9 C-terminal region protein (designated PRA, aa1651-1960,) and MYH10 C-terminal region protein (designated PRB, aa1476-2006) was produced in E.*coli* as His taged-fusion proteins (designated PRA^{His} and PRB^{His}, respectively). The purified proteins was evaluated using anti-His antibody (middle panel) and Mab2-5G2 (right panel).

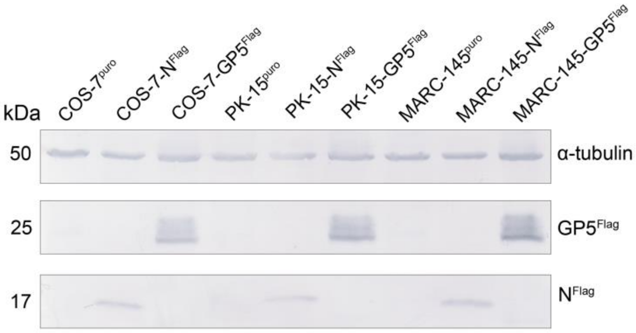

Supplementary Figure 2. Construction of cell lines stably expressing GP5^{Flag} or N^{Flag}. COS-7, PK-15, and MARC-145 cells were infected with recombinant lentivirus derived from pTRIP-GP5^{Flag}-puro, pTRIP-N^{Flag}-puro, or pTRIP-puro. After puromycin selection and subcloning, COS-7-GP5^{Flag}, COS-7-N^{Flag}, COS-7^{puro}, PK-15-GP5^{Flag}, PK-15-N^{Flag}, PK-15^{puro}, MARC-145-GP5^{Flag}, MARC-145-N^{Flag}, and MARC-145^{puro} cell lines were confirmed for proper expression patterns using western blotting with anti-FLAG and anti-α-tubulin antibodies.

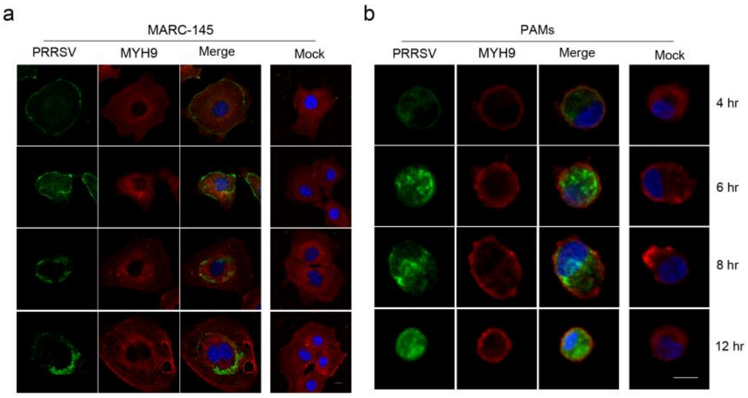

Supplementary Figure 3. PRRSV and MYH9 location in PRRSV infected cells. (a, b) The continuous assay results of Figure 2a and Figure 2b [MYH9 (red), PRRSV (green)].

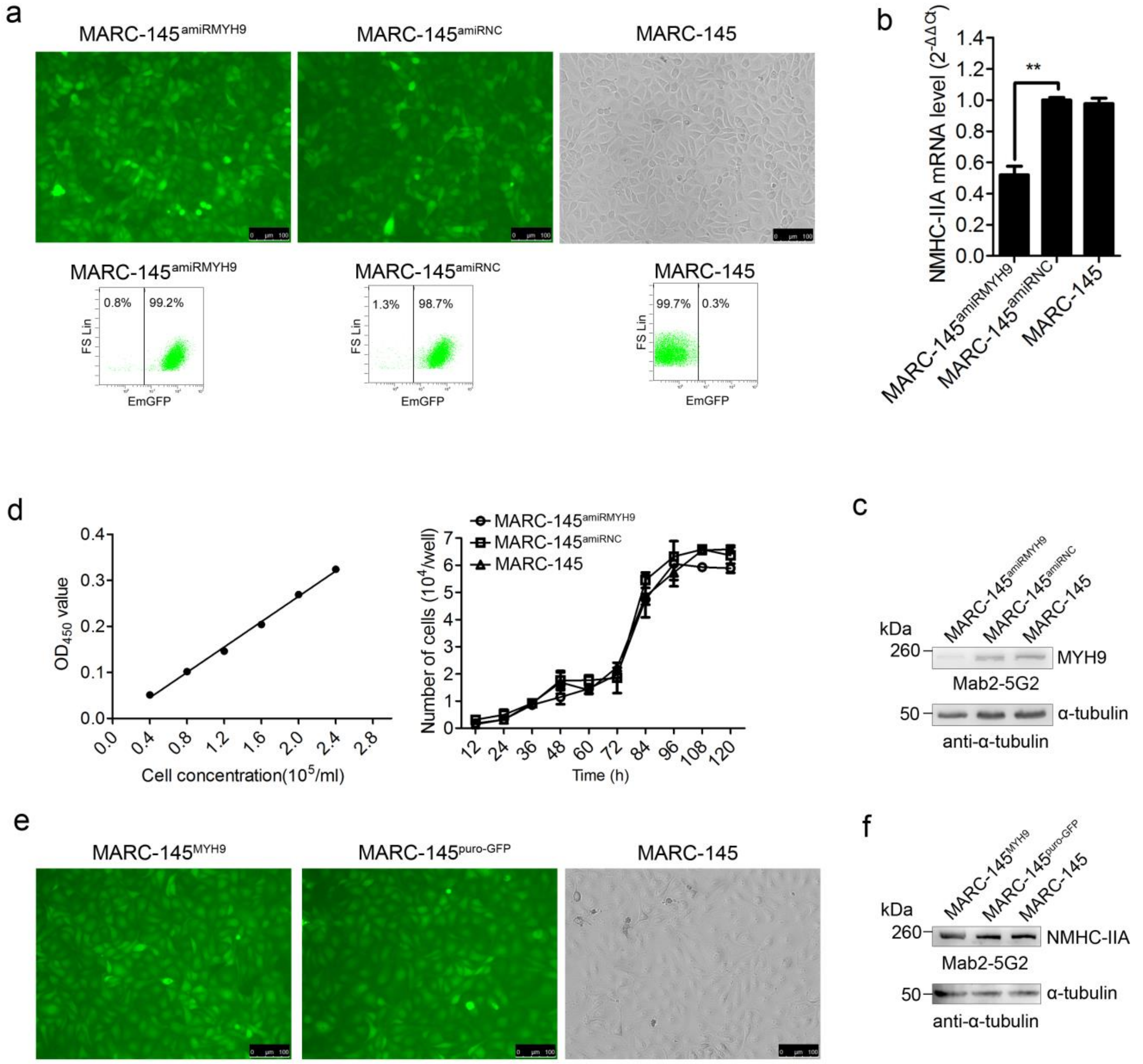

Supplementary Figure 4. Construction of MARC-145 cell lines with a knockdown or overexpression of MYH9. (a) The artificial microRNA expression plasmid pcDNA6.2-amiR^{MYH9}-EmGFP or pcDNA6.2-amiR^{NC}-EmGFP was used to transfect MARC-145 cells. After the selection using blasticidin and sub-cloning, the MARC-145^{amiRMYH9} cell line with stably reduced MYH9 expression, and the control MARC-145^{amiRNC} cell line were constructed. Flow cytometry analysis showed that more than 98% of cells were EmGFP positive. Scale bars, 100μm. Levels of MYH9 mRNA **(b)** and protein **(c)** in MARC-145^{amiRMYH9} cell line were reduced significantly than that in the control cell lines. Error bars represent mean ± s.e.m. (n=3). $^{**}P<0.01$. **(d)** Cell viability of the

MARC-145^{amiRMYH9} cell line was determined using the WST-8 system. The calibration curve between absorbance and cell count was set at Slope=0.1322 and R^2=0.995. The growth curves of MARC-145, MARC-145^{amiRNC}, and MARC-145^{amiRMYH9} cells were calculated based on the calibration curve. Error bars represent mean ± s.e.m. (n=3). Data were analyzed using analysis of variance (ANOVA) followed by post-test. **(e)** The MYH9 expression plasmid PB-MYH9-GFP+puro was transfected into MARC-145 cells to generate the MARC-145^{MYH9} cell line stably overexpressing MYH9. MARC-145 and MARC-145^{puro} cell lines were used as the controls and cells were detected using florescent or light microscopy. Scale bars, 100μm. **(f)** The MYH9 protein levels in MARC-145^{MYH9}, MARC-145, and MARC-145^{puro} cell lines were detected by western blotting using Mab2-5G2.

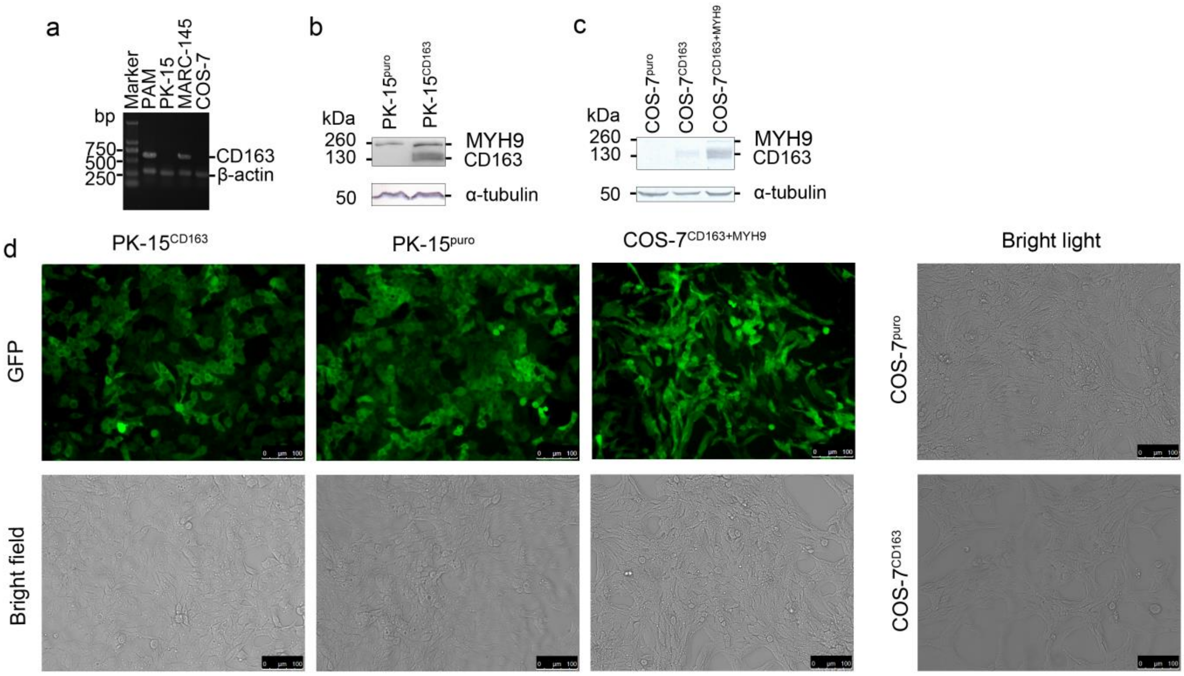

Supplementary Figure 5. Construction of PK-15^{CD163}, COS-7^{CD163}, and COS-7$^{CD163+MYH9}$ cell lines. (a) PK-15 and COS-7 cells were confirmed as lacking the CD163 gene using primers 22 and 23 listed in Supplementary Table 1 to amplify a 620bp fragment of swine CD163 (GenBank ID: JX292263) and Chlorocebus aethiops CD163 (GenBank ID:

JF753553). As the positive controls, CD163 was detected in PAMs and MARC-145 cells. β-actin gene was amplified as a reference gene using the primer 24 and 25 listed in Supplementary Table 1. **(b, c)** MYH9 and CD163 proteins in PK-15, PK-15^{CD163}, COS-7, COS-7^{CD163}, and COS-7^{puro} cell lines were detected by western blotting using Mab2-5G2 and mouse monoclonal anti-CD163 antibodies; α-tubulin was used as the loading control. **(d)** GFP florescence was observed in PK-15^{CD163}, PK-15^{puro}, and COS-7$^{CD163+MYH9}$ cell lines under the florescence microscope. COS-7^{CD163} and COS-7^{puro} cell lines were observed under the light microscope. Scale bars, 100μm.

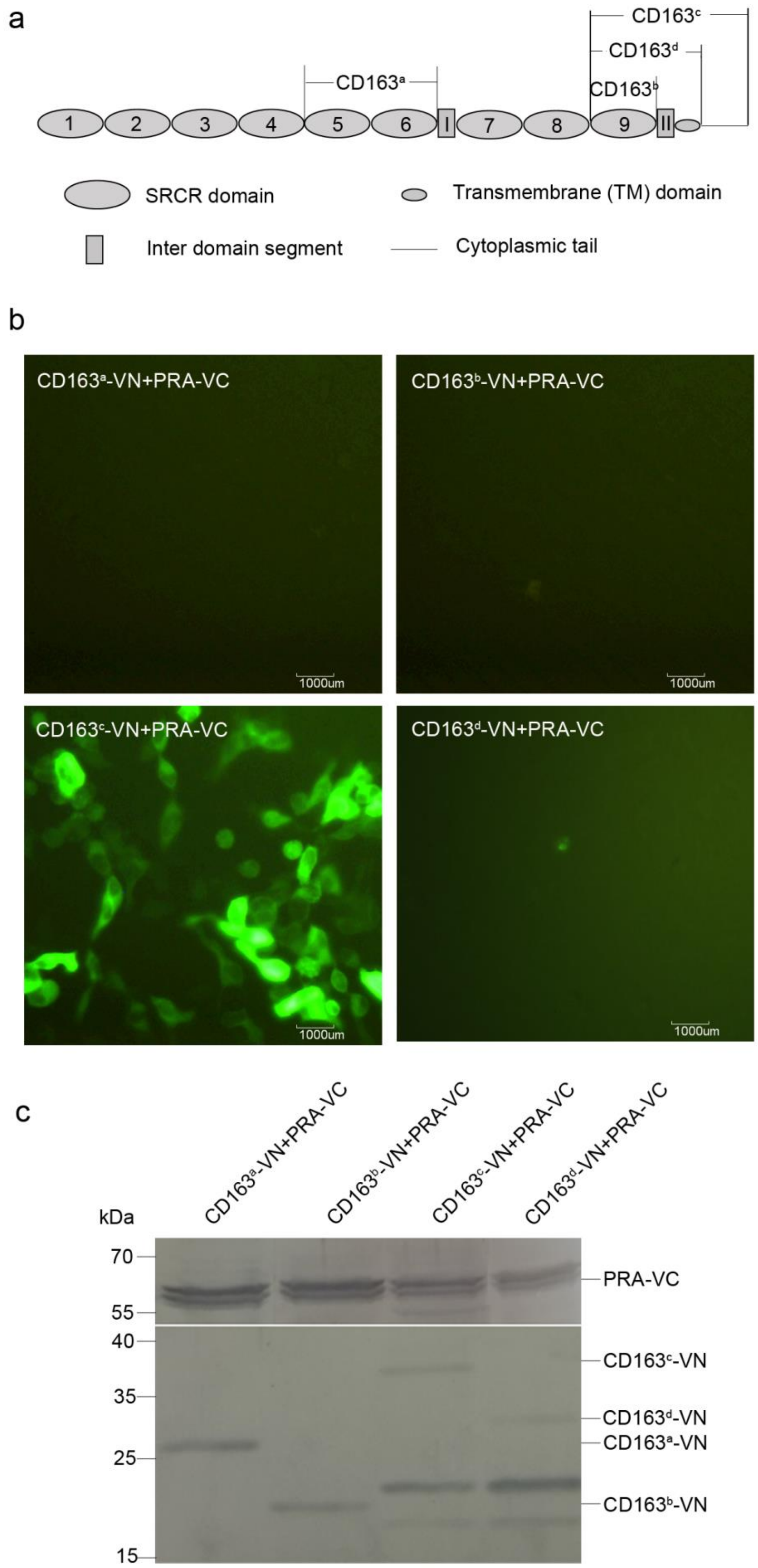

136

137 **Supplementary Figure 6.** Interaction between MYH9 C-terminal region protein (PRA) and

CD163 fragments using BiFC system. (**a**) Schematic representation of the various CD163 fragments used in this study. (b) 293T cells were transiently transfected with a combination of the indicated plasmids and incubated for 20 hrs. The fluorescence emission was detected using a fluorescence microscope. (c) After 20 hrs incubation, the cells were lysed to test the expression of PRA and CD163 fragments using Mab2-5G2 or rabbit anti-GFP polyclonal antibodies using western blot.

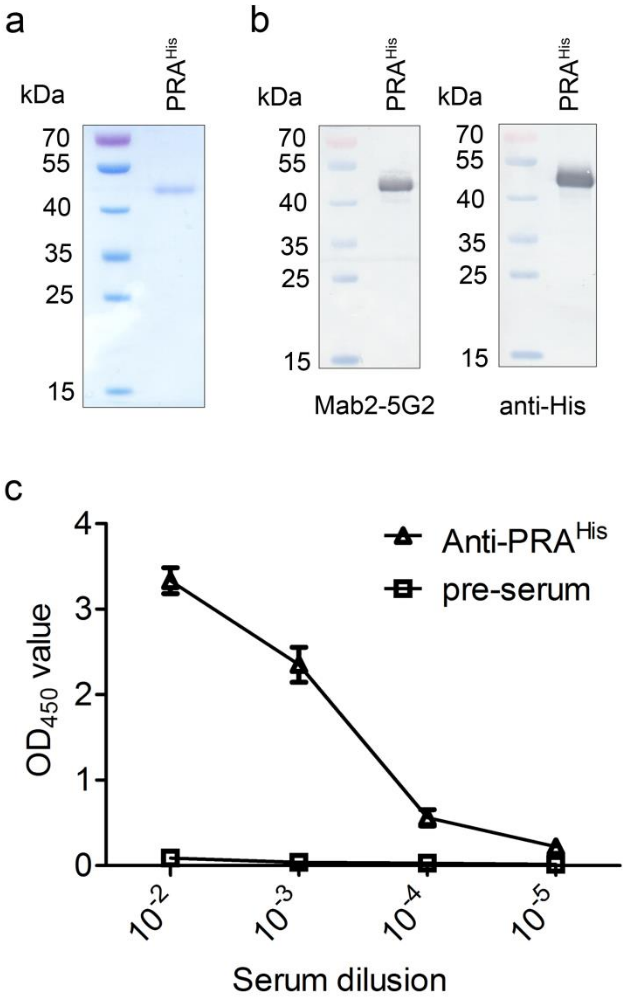

Supplementary Figure 7. Generation of rabbit anti-PRA^{His} serum. The PRA^{His} expression plasmid pET28a-PRA^{His} was transferred to *E.coli* strain BL21 and induced by IPTG (1.0 mmol/L) at 37 ℃ for 6 hrs. **(a)** PRA^{His} protein was purified by High-affinity Ni column (L00250, GenScrip Corporation, China). **(b)** The expression of PRA^{His} was identified by

western blotting using Mab2-5G2 and an anti-His tag antibody. **(c)** The purified PRA^{His} mixed with QuickAntibody-Rabbit-8W (KangBiQan, China) adjuvant was used to immunize rabbits by subcutaneous immunization (50μg/rabbit). Indirect ELISA was used to titrate rabbit anti-PRA^{His} sera with purified PRA^{His} coated on the solid-phase of ELISA plates. Goat anti-rabbit IgG were used as the secondary antibody. Error bars represent mean ± s.e.m. (n=3).

Supplementary Tables

Table S1. Oligonucleotides used in the study

Primers	Sequence (5'-3')[i, ii, iii]
1. pET28a-EcoR I-PRA^{His}-F	CCGGAATTCATGCGCGAGCTGGATGACAC
2. pET28a-Hind III-PRA^{His}-R	CCCAAGCTTTTCGGCAGGTTTGGCCTCAG
3. pET28a-EcoR I-PRB^{His}-F	TAGGAATTCGCAGAAGAGAAGAGCATC
4. pET28a-Hind III-PRB^{His}-R	CCCAAGCTTTTACTCTGACTGGGGTGGC
5. pTRIP-Nhe I-N^{Flag}-F	CTAGCTAGCATGCCAAATAACAACGGCAA
6. pTRIP-Nhe I-GP5^{Flag}-F	TAAGCTAGCATGTTGGGGAAGTGCTTG
7. N^{Flag} oligo	***AGTGCGGCCGCTCATTTATCATCATCATCTTTGTAATC***TGCTGAGGGTGATGCTGTGGCGCG
8. GP5^{Flag} oligo	***AGTGCGGCCGCTCATTTATCATCATCATCTTTGTAATC***GAGACGACCCCATTGTTCCGC
9. pTRIP-BamH I-GP5^{Flag}/N^{Flag}-R	GGCGGATCCTCATTTATCATCATCATC
10. pTRIP-CD163-In-fusion-F	*GCTGGCTAGCTCTAGA*ATGGTGCTACTTGAAGACTCTGG
11. pTRIP-CD163-In-fusion-F	*TGCAGATATCGAATTC*TCATTGTACTTCAGAGTGGTCTCCT
12. PB-MYH9-In-fusion-F	*AGATTCTAGAGCTAGC*ATGGCACAGCAAGCTGCC
13. PB-MYH9-In-fusion-R	*ATTCGAATTCGCTAGC*TTATTCGGCAGGTTTGGCC
14. Real-time-PRRSV-N-F[4]	AGATCATCGCCCAACAAAAC

15. Real-time-PRRSV-N-R[4]	GACACAATTGCCGCTCACTA
16. Real-time-MYH9-F	GACCAGATCAACACCGACCT
17. Real-time-MYH9-R	ACTTGACTGTGCCCTCCATC
18. Real-time-CD163-F[4]	TTCACTGCACTGGGACTGAG
19. Real-time-CD163-R[4]	AGGACAGTGTTTGGGACTGG
20. Real-time-β-actin-F[4]	TCCCTGGAGAAGAGCTACGA
21. Real-time-β-actin-R[4]	AGCACTGTGTTGGCGTACAG
22. CD163-detect-F	GAATGTGGAAGTGCTGTCAGT
23. CD163-detect-R	AACCACATCAGCTTCTTTCA
24. β-actin-detect-F	CAACTGGGACGACATGGAGA
25. β-actin-detect-R	GGCACAGTGTGGGTGACCCCG
26. siRNA-MYH10 (A)	GAGAAGAAGCUGAAAGAAAUC
27. siRNA-MYH10 (AS)	UUUCUUUCAGCUUCUUCUCAG
28. siRNA-negative control (A)	GAUUUAGCUAUUCAAAGAAAC
29. siRNA-negative control (AS)	UUCUUUGAAUAGCUAAAUCUU
30. PRA-VC-F	GATGGATCCATGCGCGAGCTGGAT
31. PRA-VC-R	CAGGTCGACTCATTCGGCAGGTTT

32. CD163[a]-VN-F	TATGGATCCATGCCCAGGCTGGTTGGA
33. CD163[a]-VN-R	TATAAGCTTTCATTGACCACTCCCTATG
34. CD163[b]-VN-F	CGCGGATCCATGATAAGACTTCAAGAA
35. CD163[b]-VN-R	TATGCGGCCGCGCACGTCACAGCAGCAT
36. CD163[c]-VN-R	TATGCGGCCGCTCATTGTACTTCAGAGTGGT
37. CD163[d]-VN-R	TATAAGCTTCTATTGTACTTCAGAGTGG

[i] underline is the enzyme binding site.

[ii] bold italic is tag sequence.

[iii] underline italic is the sequence in-fusion with vector.
